# Crosstalk between Receptor and Non-receptor Mediated Chemical Modes of Action in Rat Livers Converges through a Dysregulated Gene Expression Network at Tumor Suppressor Tp53

**DOI:** 10.3389/fgene.2017.00157

**Published:** 2017-10-24

**Authors:** Karen M. Funderburk, Scott S. Auerbach, Pierre R. Bushel

**Affiliations:** ^1^Department of Biology and Department of Mathematics & Statistics, College of Arts & Sciences, University of North Carolina at Greensboro, Greensboro, NC, United States; ^2^Microarray and Genome Informatics Group, Biostatistics and Computational Biology Branch, National Institute of Environmental Health Sciences, Durham, NC, United States; ^3^Toxicoinformatics Group, Biomolecular Screening Branch, National Institute of Environmental Health Sciences, Durham, NC, United States

**Keywords:** mode of action, gene expression, gene network, crosstalk, chemicals, toxicants, WGCNA, toxicogenomics

## Abstract

Chemicals, toxicants, and environmental stressors mediate their biologic effect through specific modes of action (MOAs). These encompass key molecular events that lead to changes in the expression of genes within regulatory pathways. Elucidating shared biologic processes and overlapping gene networks will help to better understand the toxicologic effects on biological systems. In this study we used a weighted network analysis of gene expression data from the livers of male Sprague-Dawley rats exposed to chemicals that elicit their effects through receptor-mediated MOAs (aryl hydrocarbon receptor, orphan nuclear hormone receptor, or peroxisome proliferator-activated receptor-α) or non-receptor-mediated MOAs (cytotoxicity or DNA damage). Four gene networks were highly preserved and statistically significant in each of the two MOA classes. Three of the four networks contain genes that enrich for immunity and defense. However, many canonical pathways related to an immune response were activated from exposure to the non-receptor-mediated MOA chemicals and deactivated from exposure to the receptor-mediated MOA chemicals. The top gene network contains a module with 33 genes including tumor suppressor TP53 as the central hub which was slightly up-regulated in gene expression compared to control. Although, there is crosstalk between the two MOA classes of chemicals at the TP53 gene network, more than half of the genes are dysregulated in opposite directions. For example, Thromboxane A Synthase 1 (*Tbxas1*), a cytochrome P450 protein coding gene regulated by Tp53, is down-regulated by exposure to the receptor-mediated chemicals but up-regulated by the non-receptor-mediated chemicals. The regulation of gene expression by the chemicals within MOA classes was consistent despite varying alanine transaminase and aspartate aminotransferase liver enzyme measurements. These results suggest that overlap in toxicologic pathways by chemicals with different MOAs provides common mechanisms for discordant regulation of gene expression within molecular networks.

## Introduction

The environment that humans and other species are exposed to is a complex space that contains various biologic stressors (natural and manufactured) which can alter cellular processes and in some cases, result in disease and affliction (Wild, 2005; Rappaport and Smith, 2010). Toxicants elicit their toxicologic effect in the liver through an engagement of target macromolecules which leads to a cascade of events referred to as modes of action (MOAs; Casarett et al., 2001). Genomic signatures manifested from toxicant exposure reflect the gene regulation that orchestrates downstream signaling through a particular MOA (Nijman, 2015). Toxicants that act through different molecular initiating events possess distinct MOAs and therefore exhibit unique genomic signatures.

Several efforts have been undertaken to identify gene expression signatures in response to toxicant exposures and to classify chemicals according to their molecular fingerprints (Amin et al., 2002; Bushel et al., 2002; Hamadeh et al., 2002a,b; Kleinjans, 2014; Wei et al., 2014). There are several known cases of chemicals that exert their effect through a particular MOA and have overlaps in the gene expression regulatory networks that regulate the biological processes. For instance, nuclear receptor-mediated chemicals such as those that act through the aryl hydrocarbon receptor (AhR), the peroxisome proliferator-activated receptor (PPAR), or the constitutive androstane receptor and pregnane X receptor (CAR/PXR) have a high degree of agreement between the molecular pathways that are perturbed (Woods et al., 2007). However, little is known about the overlapping regulatory pathways between toxicants that exert their effect through different MOAs.

Reconstruction of gene regulatory networks from gene expression data has assisted in resolving connections between genes during static conditions or dynamically as conditions change over time, dose concentration and/or target tissue (Karlebach and Shamir, 2008). Comparing gene regulatory networks to identify overlaps in connected regions is a challenge. The weighted gene correlation network analysis (WGCNA) method is designed to resolve preserved co-expression gene network modules between two conditions (Zhang and Horvath, 2005; Yip and Horvath, 2007). The approach uses transformations of the correlation between co-expressed genes to reveal interconnectedness amongst gene network nodes and permutation procedures to identify statistically significant gene network modules that overlap between sample conditions (Langfelder et al., 2011). Recent utilization of WGCNA on rat liver gene expression data from drug toxicity studies revealed 415 gene network models that associate with mechanisms of liver pathogenesis (Sutherland et al., 2017). We used the WGCNA approach to reconstruct gene networks using microarray gene expression data from male rat livers and identify preserved modules between chemicals that exert their MOA through receptors (RM) vs. those that are non-receptor-mediated (NRM). We found that the most significant gene network contains 33 genes including tumor suppressor TP53 as the central hub and that the majority of the genes were regulated in opposite directions between the RM and NRM samples. Although there is crosstalk between the two MOA classes of chemicals at the Tp53 signaling pathway, more than half of the genes are dysregulated in opposite directions. The read across between gene networks of chemicals with different MOAs suggests flexibility in the regulatory components of molecular systems to utilize common gene networks to maximize diversity in biological responses.

## Material and methods

### Chemicals and modes of action

Fifteen chemicals, each with a given dose and duration of exposure, were used for this study (Table 1). Sets of three chemicals share one of five MOAs. Three MOAs are associated with well-defined RM processes: peroxisome proliferator-activated receptor-α (PPARA), orphan nuclear hormone receptors (CAR/PXR), and aryl hydrocarbon receptor (AhR). The other two are NRM: DNA damage (DNA_damage) and cytotoxicity (Cytotox). The chemicals were administered orally or by intraperitoneal, intravenous or subcutaneous injection (5 ml/kg body weight). In order to ensure a maximal transcriptional response, 5-day maximum tolerated doses (MTD) of the chemicals were administered to the study animals. The MTD was determined in a 5-day dose range-finding study in which an MTD was determined as a 5–10% reduction in body weight relative to control.

**Table 1:** Chemical exposures and modes of action.

**MOA**	**Chemical**	**Dose (mg/kg body weight)**	**Duration (days)**
Aryl hydrocarbon receptor (AhR)	3-Methylcholanthrene (3ME)	300	5
	Leflunomide (LEF)	60	5
	beta-Naphthoflavone (NAP)	1,500	5
Orphan nuclear hormone receptors (CAR/PXR)	Phenobarbital (PHE)	54	5
	Methimazole (MET)	100	3
	Econazole (ECO)	334	5
Cytotoxicity (Cytotox)	Chloroform (CHO)	600	5
	Thioacetamide (THI)	200	5
	Carbon tetrachloride (CAR)	1,175	7
DNA Damage (DNA_Damage)	Aflatoxin B1 (AFL)	0.3	5
	Ifosfamide (IFO)	143	3
	N-Nitrosodimethylamine (NIT)	10	5
Peroxisome proliferator-activated receptor alpha (PPARA)	Pirinixic acid (PIR)	364	5
	Bezafibrate (BEZ)	617	7
	Nafenopin (NAF)	338	5

### Microarray gene expression data

Total RNA extracted from the livers of male Sprague-Dawley rats exposed once daily for 3, 5, or 7 days in triplicate, depending on the chemical or vehicle control (saline, corn oil or carboxymethyl cellulose), were processed for microarray analysis as previously described (Wang et al., 2014). Animals were handled in accordance with The United States Department of Agriculture and Code of Federal Regulations Animal Welfare Act (9 CFR Parts 1, 2, and 3). Ethics committee approval was not required according to the local and national guidelines. Fragmented cRNA prepared from liver RNA was labeled and hybridized to the Affymetrix whole genome GeneChip® Rat Genome 230 2.0 Array comprised of 31,099 gene probe sets. Pixel intensity data was acquired by scanning of the arrays using the GeneChip® Scanner 3000 7G. CEL files were generated using the GCOS software. The pixel intensity data was preprocessed using the robust multichip average (RMA) algorithm (Irizarry et al., 2003a,b) which includes background correction, quantile normalization, and summarization by the median polish approach and then log base 2 transformed. Due to a batch effect in the study design, the data was preprocessed further by mean centering on the route of administration of the chemicals. Next, we performed principal component analysis (PCA)-based gene probe filtering on the preprocessed data using the Bioconductor package “pvac,” where the filtering is based on a score measuring consistency among probes within a probe set (Lu et al., 2011). The maximum value of the threshold for the score is set at 0.5, which corresponds to 50% of the total variation accounted for by the 1st principal component. Finally, the preprocessed data was converted to log base 2 ratios by subtracting the average of the controls from the treated samples matched according to nutritional status of the vehicle and route of administration (i.e., non-nutritional-intraperitoneal, intravenous or subcutaneous injection; nutritional-oral; non-nutritional-oral). The raw data is available in the Gene Expression Omnibus (GEO) database (Edgar et al., 2002; Barrett et al., 2013) under the accession number GSE47792.

### Clinical chemistry

Clinical chemistry evaluations of blood serum samples were performed using a Roche Cobas Fara chemistry analyzer (Roche Diagnostic Systems, Westwood, NJ, USA) to numerically measure enzyme levels and metabolic entities.

### Statistical modeling

The preprocessed log base 2 ratio microarray gene expression data comprised of 12,288 gene probe sets was analyzed with the following analysis of variance (ANOVA) model to identify gene probes that vary by MOA:

(1) $$\begin{array}{r} {\text{Y}}_{\text{ijklm}}=\mu +{\text{M}}_{\text{i}}+{\text{V}}_{\text{j}}+{\text{R}}_{\text{k}}+\text{D}{\left({\text{V}}^{*}\text{R}\right)}_{\text{jkl}}+\varepsilon_{\text{ijklm}} \end{array}$$

where Y_ijklm_ represents the m^th^ observation on the i^th^ MOA (M), j^th^ vehicle (V), k^th^ route (R) and l^th^ study date (D). μ is the common effect for the whole study and ε_ijklm_ represents the random error. The errors ε_ijklm_ are assumed to be normally and independently distributed with mean 0 and standard deviation δ for all measurements. Significant gene probes that vary according to the MOAs were detected at a Benjamini–Hochberg false discovery rate (FDR) < 0.01.

### Weighted gene correlation network analysis

The log base 2 ratio data of the 2,930 gene probe sets (2,405 genes) that vary significantly according to MOAs were averaged by replicate chemicals then divided into two data sets based on the manner in which the chemicals elicit their toxic effect: RM (AhR, CAR/PXR, and PPARA) and NRM (Cytotox and DNA_damage). A gene network was reconstructed for each data set using the WGCNA method (Zhang and Horvath, 2005; Yip and Horvath, 2007; Langfelder and Horvath, 2008). Briefly, a similarity matrix **S** is generated for each data set to determine how similar in expression genes are. Here, **S** is comprised of the Pearson correlation of the *i*th and *j*th gene probe sets (*s*_*ij*_) within a data set. Then, **S** is transformed to an adjacency matrix **A** to ascertain groups of co-expressed genes. Here we used the following soft power adjacency function to generate **A**:

(2) $$\begin{array}{r} a_{ij}=|s_{ij}{|}^{\beta } \end{array}$$

where β ≥ 1 is a user defined power parameter to control the thresholding of the grouping of the co-expressed genes. The higher the value of β, the fewer co-expressed genes are grouped together. We set β = 10. Finally, a determination is made if two nodes of co-expressed genes overlap. The topological overlap matrix (TOM) Ω measures two nodes interconnectedness and is computed as:

(3) $$\begin{array}{r} w_{ij}=\frac{|N_{1}\left(i\right)\cap N_{1}\left(j\right)|+a_{ij}}{\min\left\{\left|N_{1}\left(i\right)|,|N_{1}\left(j\right)\right|\right\}+1-a_{ij}} \end{array}$$

where N_1_(*i*) denotes the set of direct neighbors of node *i*, |·| denotes the number of elements (i.e., the cardinality) and |_*N*_1_(*i*) ∩ *N*_1_(*j*)|_ denotes the number of neighbor nodes that *i* and *j* have in common. Note that *w*_*ij*_ is bounded between 0 and 1: *w*_*ij*_ = 0 if nodes *i* and *j* are not connected and the two nodes do not share any neighbors; *w*_*ij*_ = 1 if there is a direct link between the two nodes and if one set of direct neighbors is a subset of the other. The topological dissimilarity measure is denoted as

(4) $$\begin{array}{r} d_{ij}^{w}=1-w_{ij}. \end{array}$$

Significance of preserved co-expressed genes network modules between RM and NRM exposures is determined by a permutation based composite Z statistic (Z_summary_) defined as the mean of Z scores computed for density and connectivity measures (Yip and Horvath, 2007; Langfelder et al., 2011).

## Results

### Chemicals grouped by mode of action

To investigate the gene regulatory crosstalk between RM and NRM chemicals, we used the microarray gene expression data recently published from the livers of male Sprague-Dawley rats exposed in triplicate to various chemicals with different MOAs (Wang et al., 2014). The chemicals and their MOAs are listed in Table 1 along with the doses and durations of exposure. Each MOA consists of 3 chemicals. The five MOAs are mediated by aryl hydrocarbon receptor (Ahr), orphan nuclear hormone receptors (CAR/PXR), cytotoxicity (Cytotox), DNA damage (DNA_Damage) or peroxisome proliferator-activated receptor-α (PPARA). Clinical chemistry analysis of the samples revealed that alanine transaminase (ALT) and aspartate aminotransferase (AST) liver enzymes levels were substantially higher from the Cytotox and PPARA chemicals than the others indicative of more marked injury to the organ (Table 2).

**Table 2:** Clinical chemistry of samples by mode of action.

**Measurement**	**NN-IP**	**NN-OG**	**NU-OG**	**AhR**	**CAR/PXR**	**PPARA**	**Cytotox**	**DNA damage**
ALT (U/L)	45.50	45.67	69.00	33.44	55.63	93.33	273.83	54.00
	11.94	7.55	13.81	9.79	13.33	41.73	106.02	9.70
AST (U/L)	80.50	76.50	84.80	69.56	72.44	155.67	456.00	98.50
	13.11	9.14	10.91	16.76	25.23	104.18	217.18	20.23
Albumin (g/L)	3.90	3.73	4.00	3.66	4.01	4.76	3.92	4.07
	0.17	0.33	0.11	0.47	0.27	0.24	0.18	0.08
BUN (mg/dL)	13.25	13.67	14.20	30.67	15.38	14.44	34.00	18.33
	1.32	2.80	1.47	37.01	3.94	3.24	31.86	2.16
Cholesterol (mg/dL)	73.00	68.67	70.40	117.33	76.67	68.22	66.83	67.33
	1.10	17.39	7.06	29.47	16.16	24.38	45.65	19.65
Creatine Phosphokinase (U/L)	571.00	192.33	269.20	178.78	345.56	506.67	521.67	333.83
	269.33	37.92	126.44	54.25	376.27	631.03	431.97	192.74
Glucose (mg/dL)	196.75	156.50	165.60	170.00	143.33	133.67	123.00	159.67
	20.56	29.32	5.75	28.39	14.05	10.67	11.72	10.19
Lactate Dehydrogenase (U/L)	175.00	145.67	128.20	137.00	141.22	356.67	270.50	145.50
	69.31	52.66	44.62	62.97	92.20	300.81	163.12	57.36
Total Bilirubin (mg/dL)	0.15	0.36	0.14	0.31	0.16	0.16	0.42	0.15
	0.04	0.21	0.05	0.11	0.05	0.09	0.24	0.04

*Shown in the top line of each row is the mean of the measurement for all samples within a MOA. Shown in the bottom line of each row is the standard deviation of the mean*.

**Figure 1:**
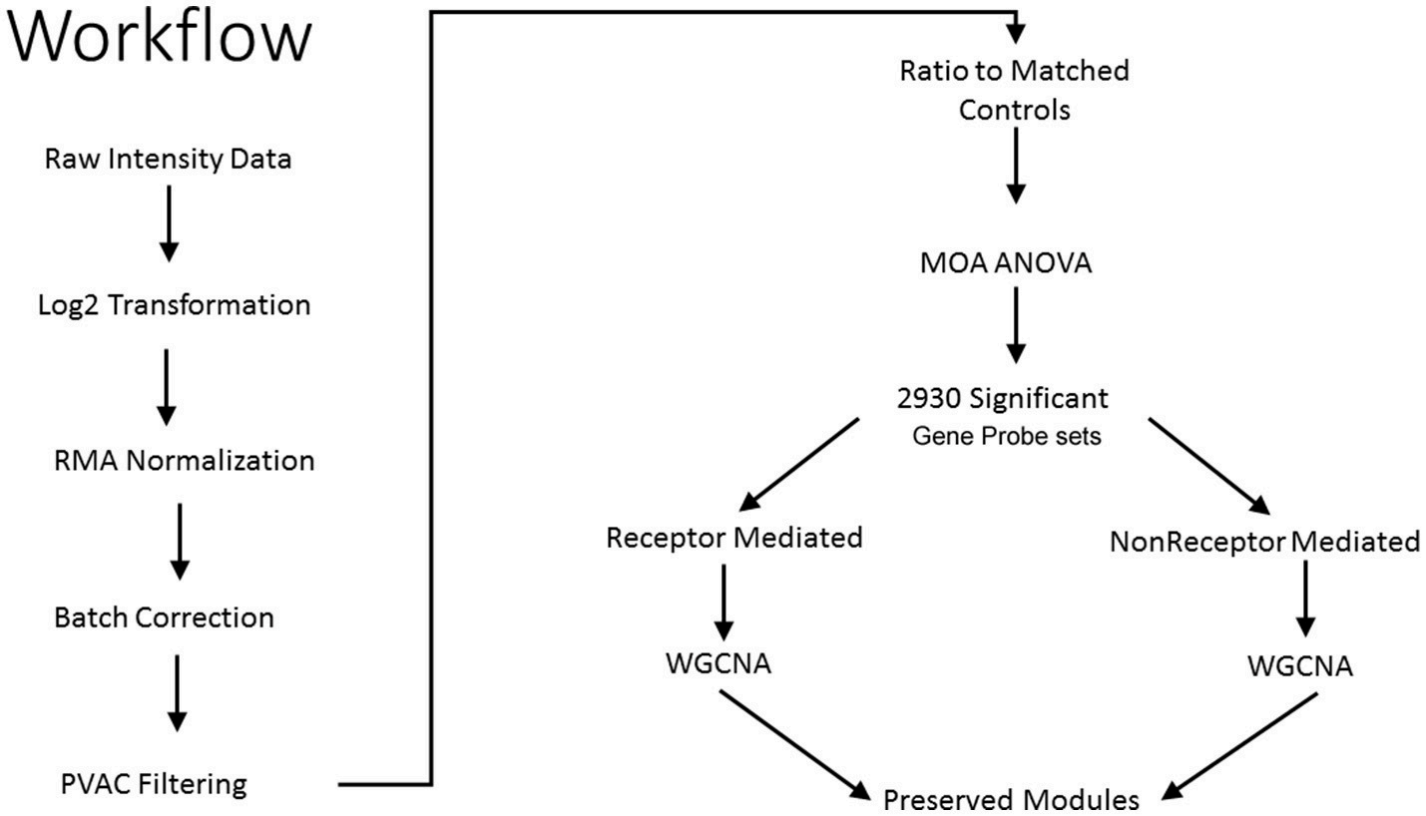
Workflow to identify preserved gene co-expression network modules. Illustrated is the analytical workflow to preprocess the microarray gene expression data, detect significant gene probe sets, and identify preserved gene network modules between the receptor-mediated (RM) samples and the non-receptor-mediated (NRM) samples. MOA is mode of action, ANOVA is analysis of variance and WGCNA is weighted gene correlation network analysis.

**Figure 2:**
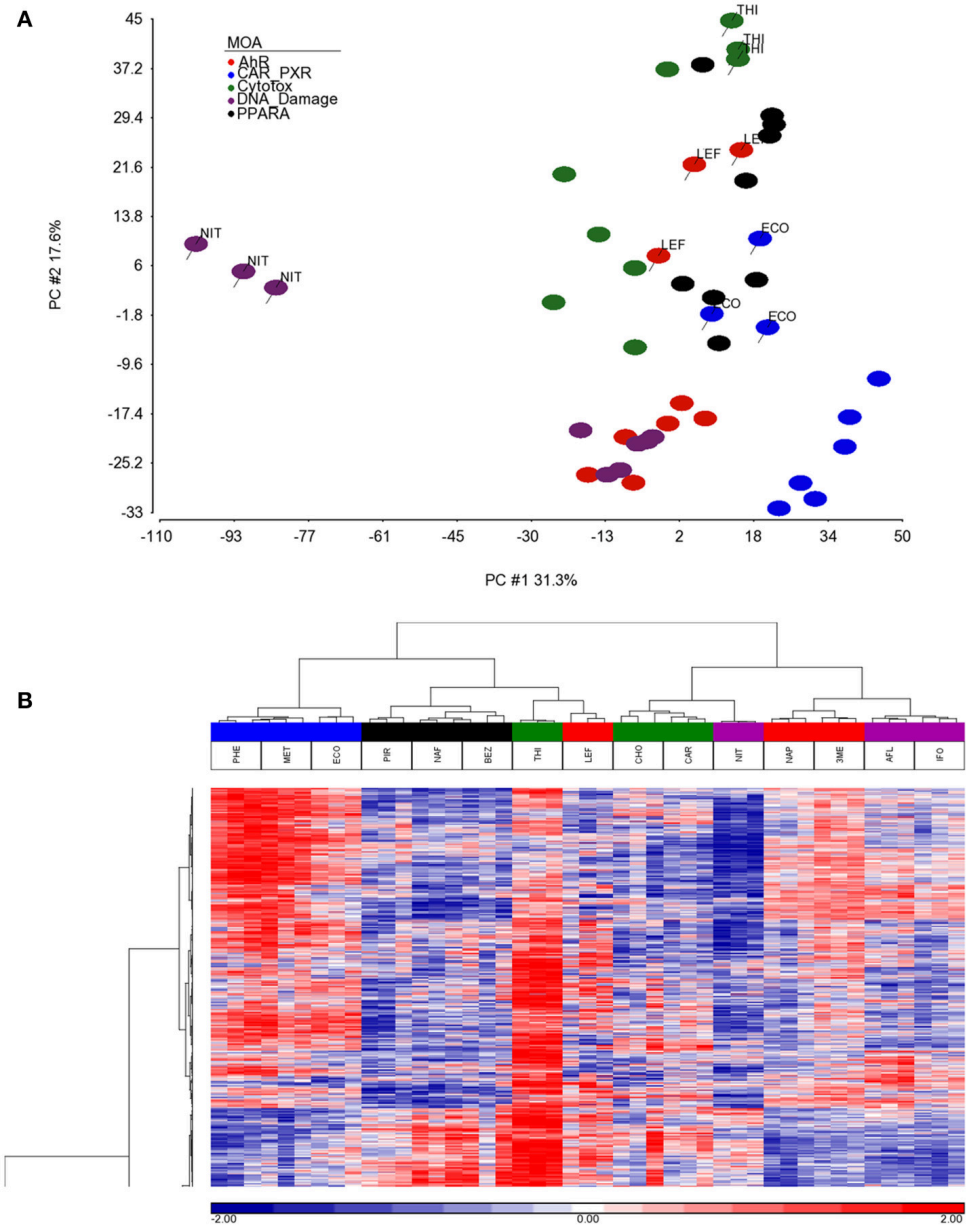
Separation and clustering of samples exposed to the chemicals in triplicate. **(A)** Principal component analysis (PCA) separation of the MOA samples using the 2,930 significant gene probe sets that vary by MOA. The x-axis is PC#1 (31.3% variation captured), the y-axis is PC#2 (17.6% variation captured) and the colors represent the MOAs as shown in the figure legend. **(B)** Two-dimensional hierarchical, agglomerative clustering of the MOA samples using the 2,930 significant gene probe sets that vary by MOA. Clustering performed using Spearman rank as the similarity metric and the Ward minimum variance criterion for grouping. The x-axis is the MOA samples colored as described in the legend to **(A)**, the y-axis is the 2,930 significant gene probe sets. The data is the log base 2 ratio (treated sample to the average of the controls matched according to nutritional status of the vehicle and route of administration) and the scale on the bottom displays the color range for the log base 2 ratio values standardized to mean 0 and standard deviation of 1. Red denotes up-regulation, blue down-regulation, and white relatively no change.

As shown in Figure 1, we use a bioinformatics workflow to process the gene expression data for statistical analysis. Following the preprocessing and filtering of the data, we modeled it with a MOA-ANOVA to identify 2,930 gene probe sets that vary statistically according to one or more of the MOAs. Using these dysregulated genes to project the samples into two-dimensional space by the amount of variability captured in principal components 1 and 2 (PC#1 and PC#2), we see that although the majority of the chemicals within each MOA grouped close to each other, four chemicals (NIT, THI, ECO, and LEF) are separated from the other chemicals that are in their respective MOA (Figure 2A). The NIT samples are separated far from all other samples possibly because they were the only ones that exhibited a high level (moderate severity) of centrilobular necrosis of the liver from the exposure (Data not shown). The THI treated liver samples exhibited minimal centrilobular necrosis in all three replicates (Data not shown). This departure from the cohesiveness of the grouping of the chemicals within their MOA is also observed in the hierarchical clustering of chemicals by MOA into two branches of the dendrogram (Figure 2B). RM chemicals in MOAs CAR/PXR and PPARA cluster together and NRM chemicals in MOAs Cytotox and DNA_Damage cluster together. However, the Cytotox chemical THI clusters with the RM chemicals and the NAP and 3ME chemicals clusters with the NRM chemicals. This suggests that although the chemicals share a MOA, the underlying gene expression changes elicited from the exposures can vary whether mediated by a receptor or not. Of interest is to determine if there are overlaps (i.e., read across) in gene expression between RM chemicals and NRM chemicals in the rat liver.

### Derivation of gene co-expression networks

**Figure 3:**
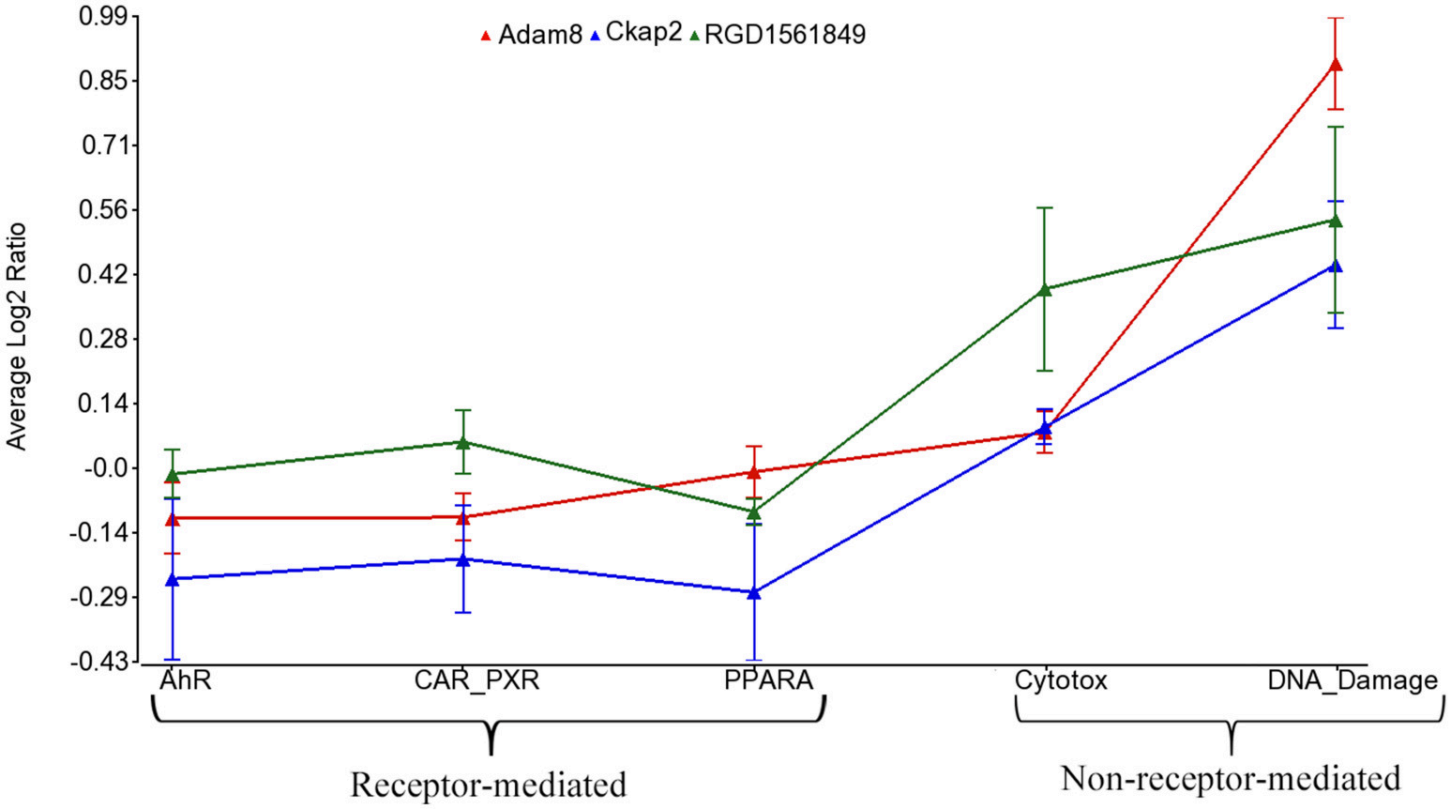
Profile plot of significantly different genes between RM and NRM samples. Gene expression profile plot of the top 3 of 17 genes determined to be statistically significant between RM and NRM samples (*p* < 0.05). The x-axis is the MOAs, the y-axis is the MOA average of the log base 2 ratio data [treated samples (the average of all three replicates for each chemical within a MOA) to the average of the controls matched according to nutritional status of the vehicle and route of administration]. The gene expression profiles are colored as shown in the figure legend. The variation in the data points from the average of the chemicals in a MOA is represented by standard error bars.

Using the 2,930 dysregulated gene probe sets, we grouped the Ahr, CAR/PXR, and PPARA chemicals into a RM class and the Cytotox and DNA_Damage chemicals into a NRM class. Table 3 lists the genes detected as statistically significant between the two classes. The expression of all the genes from exposure to the RM chemicals are down-regulated in comparison to NRM chemicals. Figure 3 shows a profile plot of the three genes (*Adam8, Ckap2*, and RGD1561849) that are down-regulated most.

**Table 3:** Differentially expressed genes between receptor-mediated and non-receptor-mediated MOAs.

**ProbeID**	**Entrez gene**	**Gene symbol**	**Gene description**	***p*-value**	**Fold change**
1392754_at	499285	Adam8	ADAM metallopeptidase domain 8	3.47E-03	−1.47
1384068_at	306575	Ckap2	Cytoskeleton associated protein 2	1.35E-03	−1.42
1390317_at	500393	RGD1561849	Similar to RIKEN cDNA 3110035E14	6.86E-04	−1.40
1370902_at	286921 /// 296972	Akr1b10	Aldo-keto reductase family 1, member B10	1.35E-02	−1.39
1368271_a_at	79451	Fabp4	Fatty acid binding protein 4, adipocyte	2.01E-02	−1.38
1374775_at	291234	Mki67	Marker of proliferation Ki-67	9.28E-03	−1.38
1383747_at	361921	Ect2	Epithelial cell transforming 2	9.89E-03	−1.37
1379582_a_at	114494	Ccna2	Cyclin A2	4.45E-02	−1.35
1384449_at	100910797	LOC100910797	Uncharacterized	4.02E-05	−1.35
1398540_at	54289	Rgs1	Regulator of G-protein signaling 1	9.50E-03	−1.35
1393041_at	362519	Smc2	Structural maintenance of chromosomes 2	4.19E-03	−1.34
1370462_at	25460	Hmmr	Hyaluronan mediated motility receptor	4.16E-03	−1.33
1384231_at	364648	Shcbp1	Shc SH2-domain binding protein 1	5.34E-03	−1.33
1393848_at	362720 /// 100359539	Rrm2	Ribonucleotide reductase M2	1.21E-02	−1.32
1383578_at	499870 /// 100911267	Rad51	DNA repair protein RAD51 recombinase	8.33E-03	−1.32
1390659_at	25406	Cd44	CD44 molecule	2.94E-02	−1.32
1371074_a_at	–	–	–	3.32E-03	−1.30

*Gene detected as statistically different in a one-way ANOVA model with the Fisher's least significant difference test between RM vs. NRM samples based on the average (triplicates per chemical) log2 ratio values of the 2,930 MOA-varying gene probe sets*.

**Figure 4:**
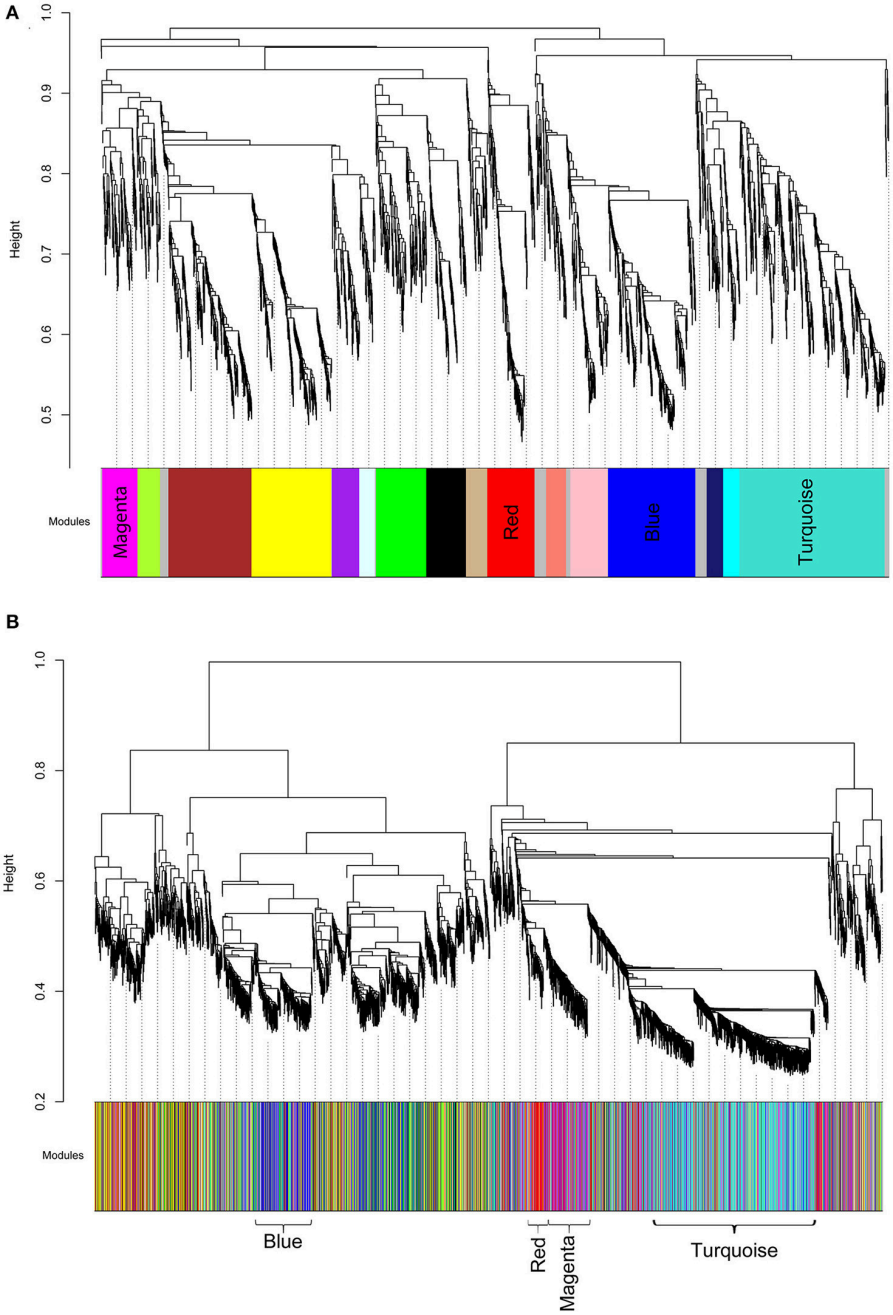
Cluster dendrograms and modules representative of gene co-expression networks. **(A)** RM cluster dendrogram. **(B)** NRM cluster dendrogram. The x-axes represent the modules identified relative to the RM clustering. The colors indicate the preserved modules. The significantly preserved modules are labeled by color. The y-axes show the heights where clusters are merged.

We then analyzed the 2,930 dysregulated gene probe sets with the WGCNA method to identify gene networks preserved between the two classes (Figure 1). Correlation between gene expression is measured by Pearson correlation and the determination of co-expression is accomplished by using an adjacency function. The interconnectedness of nodes of co-expressed genes in the network is assessed by a topology distance metric. Figure 4A depicts the RM co-expressed genes nodes as leaves in the dendrogram with the more similarly expressed genes grouped closer together. The colored modules represent the gene networks that were identified. The turquoise colored module is the largest of the 18 identified. Figure 4B illustrates the clustering of the NRM co-expressed genes nodes and the superimposing of the 18 RM modules. As can be seen by the diffuse overlapping of the modules in the two classes, the turquoise, magenta, red and blue colored ones are preserved most readily. This preservation is statistically assessed by a permutation test to derive of a summary Z score. The significant modules (Z_summary score > 10) are shown in Table 4 with turquoise being the most significant. The gene network sizes are 540, 176, 325, and 131 gene probe sets for the turquoise, red, blue, and magenta modules respectively.

**Table 4:** Preserved modules.

**Module**	**Module size**	**Z summary**
Turquoise	540	27.27
Red	176	16.93
Blue	325	15.00
Magenta	131	11.92

### Pathways and biological processes read across genes in preserved modules

**Figure 5:**
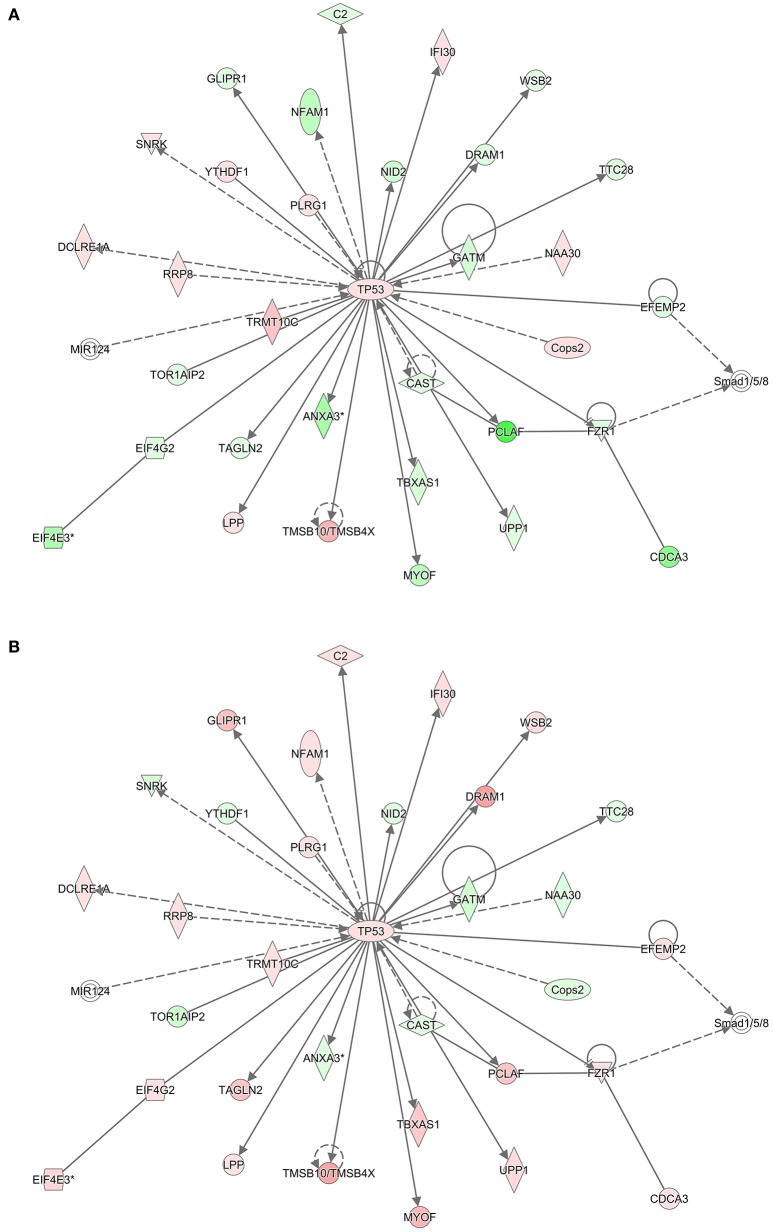
TP53 Interaction network. Using the 540 gene probe sets (462 genes) from the most significant module (Turquoise) preserved between **(A)** RM and **(B)** NRM samples that were mapped to pathways in the Ingenuity Pathway Analysis knowledgebase, molecular networks were generated. Shown is the most significant interaction network with TP53 as the central hub. Colored nodes represent 33 genes (or their products) that are part of the 540 gene probe sets. The gene expression values are the log base 2 ratio from the average of the triplicates for each chemical treatment to the average of the controls matched according to nutritional status of the vehicle and route of administration, averaged by MOA and according to either RM or NRM. Red represents increased expression and green represents decreased expression. Shape representations: circles, protein-coding genes; diamonds, enzymes; squares, cytokines; horizontal ovals, transcription regulators; vertical ovals, transmembrane receptors. A solid line represents a direct interaction, whereas a dashed line represents an indirect interaction. A line with an arrow denotes activation, whereas a line with an arrow and a pipe (|) denotes acts on and inhibits, respectively. A line without an arrow or pipe (|) denotes a protein–protein interaction.

To infer which pathways are over-represented by the genes in the preserved modules, we performed an enrichment test using the Protein ANalysis THrough Evolutionary Relationships (PANTHER) ontology database (Thomas et al., 2003). Table 5 shows the significant biological processes that were enriched by the genes in each of the preserved modules. Immunity and defense was overwhelmingly significant (FDR < 10%) by the genes in three of the four modules. Using the Ingenuity Pathway Analysis (IPA) knowledgebase, we discovered that TP53 is a central hub of the 33 genes from the turquoise module that have connections (Figure 5). Interestingly, although the connections are the same between the RM and the NRM chemicals due to the preservation of the turquoise module, the expression of more than half of the genes are dysregulated in opposite directions. Some of these genes code for proteins that are associated with cell division (FZR1, CDCA3), metabolism (TBXAS1), and DNA repair (PCLAF).

**Table 5:** Pathway enrichment.

**Module**	**Panther db ID**	**Biological process**	**Count**	**Fold enrichment**	***p*-value**	**FDR**
Turquoise	BP00148	Immunity and defense	68	2.38	1.48E-11	1.8E-08
Red	BP00148	Immunity and defense	54	1.72	8.37E-05	1.0E-01
Blue	BP00125	Intracellular protein traffic	33	1.66	4.93E-03	5.8E+00
Magenta	BP00148	Immunity and defense	52	1.66	2.73E-04	3.3E-01

## Discussion

Exposure to chemicals can elicit pharmacologic effects if therapeutic, tailored accordingly and given at the right dose for an appropriate amount of time. In other cases, the exposure can have no detectable effect or can be toxic resulting in an adverse effect to biological systems. The molecular initiating events for many chemicals are well-studied. However, their MOAs remain to be determined. Having a better understanding of a chemical's MOA and the molecular consequences from their exposure will aid in determining points of potential crosstalk between regulatory pathways which may lead to unintended side effects if chemicals act synergistically.

We used gene expression from the livers of male Sprague-Dawley rats exposed to a number of agents (Table 1) or vehicle control to identify overlapping gene networks between chemicals that are receptor-mediated (RM) and those that are non-receptor-mediated (NRM). The RM class of chemicals contained those that elicit their effect through either the peroxisome proliferator-activated receptor-α (PPARA), orphan nuclear hormone receptors (CAR/PXR) or aryl hydrocarbon receptor (AhR) while the NRM chemicals do so by cytotoxicity (Cytotox) or DNA damage (DNA_Damage). Each MOA contained 3 different chemicals with each chemical exposure in triplicate. Four gene network modules were preserved in a statistically significant manner between the two classes of chemicals (Table 4). The genes in three of the four networks over-represent immunity and defense biological processes (Table 5). DNA damage and cytotoxic chemicals are known to trigger an innate immune defense by eliciting parenchymal cell death and subsequent DAMP (Danger-associated molecular patterns) release (Srikrishna and Freeze, 2009). Notably scoring of the modules using the Nextbio Body Atlas (Data not shown) reveals that genes in all four modules are over expressed in inflammatory cells including the blood, suggesting that what may be being detected are transcripts from inflammatory infiltrates that manifest following tissue damage. Notably this is consistent with observations that co-regulation modules in the liver are related, in part to changes in cellularity (i.e., increases or decreases in certain cell types; Sutherland et al., 2017). Chemicals that act through a receptor have cascades of signaling that often attenuate cell death by inhibiting apoptosis (Mally and Chipman, 2002) therefore decreasing cell turnover. The decreased cell death may secondarily down-regulate baseline immune signaling as there is less cellular debris to clear (Rock and Kono, 2008). In addition, activation of PPAR-α and CAR/PXR have been demonstrated to down-regulate the expression of compliment and coagulation factors which may also be contributing to the decreased immune signaling seen with the RM chemicals (Kramer et al., 2003; Yadetie et al., 2003; Cariello et al., 2005; Rezen et al., 2009). Hence, it is plausible that the convergence point between these two groups of toxicologic agents occurs at a cellular level and cascades down into the molecular level where opposite effects on inflammatory signaling is observed. Although many gene expression signatures associated with toxicants likely represent cytotoxicity and cell damage, activation of an immune response is not just injury *per*-*se*, but is very much involved in repair and regeneration. The toxicant gene signatures likely reflect a genomic state in the liver during the process of the ensuing injury vs. the abating of it and beginnings of recovery and repair.

Here we show that with nine RM chemicals and six NRM chemicals, a converging point in one of the gene networks is at the tumor suppressor gene *TP53* (Figure 5). Tp53 in rats is a 391 amino acid containing phosphoprotein with an amino-terminal transactivation motif, DNA and zinc binding sites, a tetramerization domain and an unstructured basic domain at the carboxy-terminus. TP53 regulates the cell cycle, it plays a role in apoptosis and DNA repair, and functions as a tumor suppressor. TP53 in humans is highly mutated in cancers (Olivier et al., 2010) and has been explored extensively as a potential target for cancer therapeutics (Parrales and Iwakuma, 2015). In this study of the male rat livers exposed to the RM and NRM chemicals, *Tp53* gene expression is slightly up-regulated relative to control (but not statistically significant with a large enough fold change difference). This is not surprising as a small change in the expression of a transcription factor can dramatically impact the transcriptional regulation of its target genes (Niwa et al., 2000; Rizzino, 2008). In addition, per the IPA knowledgebase molecular network (Figure 5), TP53 interacts with 32 genes in the turquoise module; 21 genes with p53 binding sites and the others have molecular relationships such as protein-protein interaction or some form of biochemical modification. Of these 32 hub genes, the majority of them (*n* = 19) are dysregulated in opposite directions in RM vs. NRM. Some of these genes function in metabolism, cell division and DNA repair. This redundancy in the gene network circuitry is thought to be contrapuntal in nature to provide organisms the flexibility to diversify function while conserving biologic resources (Komili and Silver, 2008). Examples are the coordinated gene expression regulation during seed development in *Arabidopsis thaliana* (Ruuska et al., 2002) and the crosstalk between Janus kinase-signal transducer and activator of transcription (JAK-STAT) and PPAR-α in COS-1 cells derived from monkey kidney tissue (Zhou and Waxman, 1999).

Although, the number of chemicals per RM and NRM MOAs limits the granularity in the reconstruction of the networks we ascertained as preserved between the two classes, the diversity in the types of chemicals, the varied structure activity groups, and broad therapeutic indications of the chemicals give credence to the biological interpretation of the molecular pathways in common but coordinately dysregulated. Furthermore, despite the incohesiveness of a few of the chemicals which did not cluster by gene expression with the other chemicals in their respective MOA (Figure 2), the bioinformatics processing of the data that we employed (Figure 1) was robust enough to elucidate molecular interaction networks that converge between the RM and NRM chemicals. However, caution in the interpretation of these results is prudent since we have not examined the entire scope of all the chemicals that fall into a given MOA and some chemicals are known to have multiple MOAs (Russom et al., 1997; Wenzel et al., 1997; Freidig et al., 1999). In addition, the comparison of RM and NRM MOAs is a simplistic one and each class does not cover all the RM or NRM MOAs, therefore is limited in the inference of the gene regulatory networks. Furthermore, the doses of the chemicals administered are of a single concentration and duration albeit the MTD and so essentially the preserved gene networks that we discovered are not dynamic in nature. It is important to emphasize that the gene modules described here are a starting point for MOA characterization and greater nuance will likely be required to characterize mechanistic processes associated with specific receptors (e.g., PPAR-α vs. AhR; LeBaron et al., 2014; Becker et al., 2015) or chemicals with mixed MOAs. An illustration of such nuance was shown in a recent study in which clear subgroups of chemicals in the RM class of compounds was observed (De Abrew et al., 2015). Further, it is important to note that careful consideration of the interpretive approach is necessary when evaluating MOA (Currie et al., 2014). In conclusion, our data and results provide a framework for investigators to follow-up on to possibly perturb individual components of biological pathways that read across between chemicals with different MOAs in order to better understand the consequences of environmental exposures.

## Author contributions

KF performed most of the analyses. PB conceptualized the analysis strategy, directed the analyses, performed some analyses, interpreted the results and wrote the paper. SA provided the samples that the data were generated from, interpreted the results and provided biological/toxicological context.

### Conflict of interest statement

The authors declare that the research was conducted in the absence of any commercial or financial relationships that could be construed as a potential conflict of interest.
